# The Pathology and Treatment of Erysipelas

**Published:** 1891-07-25

**Authors:** 


### THE PATHOLOGY AND TREATMENT OF ERYSIPELAS.

Although erysipelas has been known to surgeons for many centuries, it is only within the last few years that its real nature has been at all understood; and even now there is a great deal to be learnt about it. In spite of clearly laid down rules its diagnosis even is uncertain; for instance, whilst most text books describe the spreading outline of the inflamed area as abrupt and raised, Agnew says, "The scarlet blush blends imperceptibly with the colour of the healthy skin." Such a marked discrepancy of opinion would seem to show that, after all, there is a real difficulty in diagnosing the affection from erythema. When we come to causation and pathology of the disease we are still in doubt. Hüter was probably the first to find microbes under the skin of erysipelatous patients; Lukomsby described the method in which these germs spread in and around the lymphatics; Fehleven demonstrated that streptococci were almost always to be found, and he considered them the essential cause of the disease. Unfortunately, these chain-like masses cannot be distinguished microscopically from similar organisms found in pyæmia and in ordinary suppuration. Yet there is apparently little or no connection between epidemics of pyæmia and erysipelas. When one is rampant in the wards of a hospital (luckily a rare occurrence now) the other is usually absent. Cultivations of these germs have been made by many distinguished bacteriologists, and at first it seemed as if the method of growth in nutrient media showed slight but important differences; in fact that the "streptococcus erysipelatis" could be isolated and diagnosed. However, Eiselberg, Baumgarten, and Frankel have failed to obtain these differences, and consider the streptococcus associated with ordinary suppuration and that found in erysipelas as identical (Crookshank). We must remember, however, that the microscope may not be able to detect all the minute differences between kindred species of microbes, and it is quite possible for two very distinct forms to grow on agar-agar or gelatine in a similar manner. The symptoms of the disease all point to its zymotic nature; it has a definite period of incubation (usually a week or more); it passes through certain phases; and the constitutional disturbance is such as would be expected from the introduction of a poison-forming germ. That it is highly infectious there can be no doubt; both experiments and clinical experience prove this. We are, therefore, almost bound to suppose that erysipelas is caused essentially by a microbe peculiar to it, or that the peculiarity of the disease consists in the fact of the organism being introduced into the lymphatics instead of into the blood current. The latter hypothesis cannot be held, for the juices from a patient affected with erysipelas produce the same disease by whatever channel it is introduced.

Dr. Roswell Park, in the "Annals of Surgery" for May, 1891, points out that streptococci are usually found in the subcutaneous tissue and in the neighbourhood of lymphatics, whereas staphylococci (that is cocci arranged in groups and not dividing in one direction only), are common in pus from abscesses, boils, osteo-myelitis, &c., and do not choose the lymphatics. He instances the various forms of mastitis, in the more superficial of which (beginning round the nipple and in the larger ducts) streptococci are sometimes found, whereas in the deeper forms of mammary inflammation streptococci are more numerous. This has an important bearing on the disease under consideration; in erythema we have an acute condition, usually localised and not necessarily dependent on microbes at all; in fact a dilatation of the capillaries, causing a flush, which persists for some hours or days, is the condition underlying the phenomena of the disease. Consequently, the outline of the flush would fade off gradually into the surrounding parts, and owing to the free anastomosis of the blood-vessels, and the way they divide, this redness would spread equally in all directions from the wound or from the injured part. If microbes are present they are usually masses of staphylococci. In erysipelas, on the other hand, the disease is located at first in and around the capillary lymphatics; the germs, which exist in long chains (streptococci), travel and multiply in the lymphatic channels chiefly; these minute channels are tortuous in size; are often crowded with lymph corpuscles; the fluid in them is comparatively thick; and the circulation in them is slow. These anatomical facts explain the comparatively slow spread of erysipelas; its peculiarly shaped, irregular outline, and especially its defined and raised border, for the inflammatory redness and exudation follow steadily in the wake of the multiplying germs.

In the Laboratory Reports of the Royal College of Physicians of Edinburgh Drs. Wood and Ross record some interesting experiments on the spread of this disease. They found that if iodine or any irritating fluid be painted round the affected part the extension is effectually stopped. This is explained as follows: Metschnikoff found in erysipelas three distinct zones, in which three distinct processes are going on. In the outermost one the micrococci are spreading and filling the lymphatics, although to the naked eye the skin here is unaffected. Secondly, there is a region next to this where the vessels are dilated, and serum exuded. This is the raised margin. With the serum exude a number of amæboid cells, which busily eat up the micrococci. Thirdly, there is an innermost zone, where the smaller amæboid cells (called by Metschnikoff microphages) are being in their turn devoured by larger cells (macrophages).

It is obvious that the dilatation of the blood vessels is a most potent element in the natural cure of the disease, and the above-mentioned treatment with iodine acts by setting up an inflamed area outside the spreading vanguard of the disease, and so producing a supply of microphages to effect their work of destruction, and stop the onward progress of the enemy. It is important that the irritant be applied outside the boundary of the disease only; any application inside this line only withdraws blood from the part where it is most wanted. The pathology and the treatment go hand in hand, and the result is, therefore, satisfactory. Drs. Wood and Ross attribute the success of this treatment to two factors—(1) the increase of oxygen in the part induced by the greater blood supply following the irritation, streptococci being *anærobic*, and, therefore, destroyed by oxygen; and (2) the washing away of toxines by the increased blood stream.

Roger (in "Comptes rendus Société de Biologie," 3 Mai, 1890) made an experiment, which strikingly bears out the above views. He cut the sympathetic nerve going to the ear of a rabbit on one side, and then inoculated *both* ears with erysipelas. The disease ran a much more favourable course on the side on which the nerve had been severed, and on which the arteries were dilated. He attributed this result to the freer diapedesis of leucocytes on this side, and the consequent destruction of the streptococci.

In conclusion, we may consider the essential cause of erysipelas to be a microbe, the exact nature of which is still uncertain, although it belongs to the class of streptococci; this organism has an affinity for the lymphatic vessels, and is anærobic; it falls an easy prey to the amæboid cells which exist in inflamed parts, and the treatment, in accordance with this knowledge, is obviously to bring about a sufficient dilatation of blood vessels to induce diapedesis *in advance* of the spreading area of the disease to ensure a sufficient number of devouring cells and a sufficient supply of oxygen to destroy the enemy.

